# Assessment of need for hemostatic evaluation in patients taking valproic acid: A retrospective cross-sectional study

**DOI:** 10.1371/journal.pone.0264351

**Published:** 2022-02-25

**Authors:** Demi S. Post, Arian van der Veer, Olaf E. M. G. Schijns, Sylvia Klinkenberg, Kim Rijkers, G. Louis Wagner, Vivianne H. J. M. van Kranen-Mastenbroek, Paul C. P. H. Willems, Paul W. M. Verhezen, Erik A. M. Beckers, Floor C. J. I. Heubel-Moenen, Yvonne M. C. Henskens

**Affiliations:** 1 Department of Pediatric Hematology, Maastricht University Medical Center, Maastricht, The Netherlands; 2 Department of Pediatric Hematology, Amalia children’s hospital, RadboudUMC, Nijmegen, The Netherlands; 3 Department of Neurosurgery, Maastricht University Medical Center, Maastricht, The Netherlands; 4 Academic Center for Epileptology, Maastricht University Medical Center and Kempenhaeghe, Maastricht–Heeze, The Netherlands; 5 School for Mental Health and Neuroscience (MHeNS), University Maastricht (UM), Maastricht, The Netherlands; 6 Department of Pediatric Neurology, Maastricht University Medical Center, Maastricht, The Netherlands; 7 Department of Clinical Neurophysiology, Maastricht University Medical Center, Maastricht, The Netherlands; 8 Department of Orthopedic Surgery, Maastricht University Medical Center, Maastricht, The Netherlands; 9 Central Diagnostic Laboratory, Maastricht University Medical Center, Maastricht, The Netherlands; 10 Department of Hematology, Maastricht University Medical Center, Maastricht, The Netherlands; University of Pennsylvania Perelman School of Medicine, UNITED STATES

## Abstract

**Introduction:**

Valproic acid (VPA) is a frequently prescribed anti-epileptic drug. Since its introduction side effects on hemostasis are reported. However, studies show conflicting results, and the clinical relevance is questioned. We aimed to determine the coagulopathies induced by VPA in patients who undergo high-risk surgery. The study results warrant attention to this issue, which might contribute to reducing bleeding complications in future patients.

**Methods:**

Between January 2012 and August 2020, 73 consecutive patients using VPA were retrospectively included. Extensive laboratory hemostatic assessment (including platelet function tests) was performed before elective high-risk surgery. Patient characteristics, details of VPA treatment, and laboratory results were extracted from medical records.

**Results:**

46.6% of the patients using VPA (n = 73) showed coagulopathy. Mainly, platelet function disorder was found (36.4%). Thrombocytopenia was seen in 9.6% of the patients. Data suggested that the incidence of coagulopathies was almost twice as high in children as compared to adults and hypofibrinogenemia was only demonstrated in children. No association was found between the incidence of coagulopathies and VPA dosage (mg/kg/day).

**Conclusion:**

A considerable number of patients using VPA were diagnosed with coagulopathy, especially platelet function disorder. Further prospective studies are needed to confirm the need for comprehensive laboratory testing before elective high-risk surgery in these patients.

## Introduction

Valproic acid (VPA) is a commonly used anti-epileptic drug (AED). It is also prescribed as a mood stabilizer and for migraine prophylaxis [1–3]. Various side effects have been described, e.g. nausea, sedation, weight gain, and hepatic toxicity [1, 4]. More importantly, various life-threatening bleeding complications such as pulmonary and intracerebral hemorrhage are reported, which suggests a negative effect on hemostasis [5–7].

VPA has been reported to interfere with both primary and secondary hemostasis. Multiple studies found that VPA induced thrombocytopenia in 3–21% of the patients by bone marrow suppression and immune reactions against platelets [8–14]. Other coagulopathies that were found as a side effect of VPA are hypofibrinogenemia, factor XIII deficiency, acquired von Willebrand disease (VWD), and platelet function disorder [8, 9, 15, 16].

Many patients referred for high-risk surgery e.g., brain surgery or long track spinal fusion, frequently have the diagnosis of epilepsy and use VPA. Perioperative bleeding risks, resulting in an adverse outcome, should be taken into account during preoperative counseling. Although bleeding complications have been described in case reports, studies systematically investigating the effect of VPA on hemostasis and perioperative blood loss or bleeding complications are scarce [17–21].

Preoperative tapering of VPA is frequently no option due to severe drug-resistant epilepsy. Therefore, it is important to identify these concomitant coagulopathies in the preoperative phase. Anticipation of these disorders with a preoperative treatment plan may prevent surgery-associated morbidity and mortality in this group of patients.

The aim of this study is to illustrate relevant disorders in hemostasis in a single cohort of patients using VPA who undergo high-risk surgery. Moreover, potential differences in outcome between children and adults and patients with a high and low dosage VPA were assessed. Confidently, our results will lead to more awareness regarding the presence of hemostasis disorders in patients on VPA. Hopefully, this will reduce future bleeding-associated morbidity and mortality.

## Material and methods

### Study design and patients

A retrospective analysis was performed on a cohort of patients treated with VPA, who underwent elective high-risk surgery (surgery with predicted blood loss or morbidity because of a relatively small amount of blood loss e.g., neurosurgery or spinal fusion). These patients were consecutively tested on coagulopathies according to the local protocol in Maastricht University Medical Center (MUMC+) in the period between January 2012 to August 2020. The study was approved by the Medical Ethical Committee of MUMC+ (METC 2020–1595, azM/UM).

Participants were retrospectively selected by searching the laboratory information system (Labosys) with the search term Depakine (brand name of VPA) or VPA in the conclusion or a test result of light transmission aggregometry (LTA) in combination with one of the following medical specialties: neurosurgery, orthopedics, otolaryngology, internal medicine, hematology, pediatrics, or anesthesiology. Consequently, charts in the electronic medical record system were reviewed and participants were included if VPA was used at the time of blood sampling. No exclusion criteria were defined.

Patient-related information (age, gender, weight, medical history, medication, and family history), laboratory results (see paragraph laboratory tests for the description of all tests), VPA treatment-related information (dosage of VPA, duration of treatment and indication), and specialty were extracted from medical records.

### Blood sampling

Blood samples were collected according to protocol using ethylenediaminetetraacetic acid (EDTA) and 3.2% citrated blood tubes in patients who had not eaten fat-containing food for at least four hours in the preoperative phase. Patients who used non-steroidal anti-inflammatory drugs (NSAID) on occasion, discontinued this at least 7–14 days before blood sample collection. All hemostatic tests were carried out on citrated blood. All citrated blood tubes were checked on the right amount of filling, hemolysis, icterus, and lipemia. Platelet-rich plasma (PRP) was obtained by centrifugation of citrated blood at 170g for 10 min at 18°C. To prepare platelet-free plasma (PFP), citrated blood was centrifuged twice at 2,500g for 5 minutes and 10,000g for 10 minutes at 18°C.

### Laboratory tests

Hemoglobin (Hb), hematocrit (Ht), leukocytes, platelets and mean platelet volume (MPV) were measured using Sysmex XN 9000 (Sysmex Europe, Norderstedt, Germany). Activated partial thromboplastin time (aPTT; Dade Actin FSL, Siemens), prothrombin time (PT; Dade Innovin^®^ PT, Siemens), fibrinogen level (Dade Thrombin Reagent, Siemens), factor VIII activity (Dade Actin FS, Siemens, and Coagulation Factor VIII deficient plasma, Siemens), factor XIII activity (Berichrom^®^ FXIII, Siemens), VWF activity (INNOVANCE VWF Ac, Siemens) and VWF antigen (vWF Ag, Siemens) were performed on Sysmex CS2100i (Sysmex Europe, Norderstedt, Germany).

Platelet function was analyzed by the platelet function analyzer 200 (PFA-200; INNOVANCE PFA-200 System, Siemens, Marburg, Germany) using collagen and epinephrine (Dade PFACollagen/EPI) and collagen and ADP (Dade PFACollagen/ADP) test cartridges. Closure times were determined. In addition, platelet function was tested using LTA on Chrono-log 490D (Chrono-log Corporation, Havertown, United States of America). PRP was adjusted with PFP to obtain a platelet count of 250 x 10^9^/L. Platelets were activated with arachidonic acid 1 mmol/L (AA; Bio/DATA LS101297), thrombin receptor activating peptide 15 μmol/L (TRAP; Boom H8105), collagen 1 μg/mL and 4 μg/mL (COL; Chrono-log CH385), ristocetin 1.5 mg/mL (RIST; Chrono-log CH396), ADP 5 μmol/L and 10 μmol/L (Chrono-log CH384) and epinephrine 10 μmol/L (EPI; Chrono-log CH393).

Test results were interpreted using hospital reference ranges (S1 Table).

### Primary and secondary outcomes

The primary outcome of this study is the incidence of the different coagulopathies (defined as any discrepant result in laboratory hemostatic tests that might result in increased bleeding tendency). Thrombocytopenia is diagnosed if platelet counts are <150 x 10^9^/L, hypofibrinogenemia if fibrinogen level is <1.7 g/L, low VWF activity if VWF activity or antigen is 30–50%, von Willebrand disease (VWD) if VFW activity or antigen is <30%, factor VIII deficiency if the activity is <50%, factor XIII deficiency if the activity is <70% and platelet function disorder if LTA is abnormal after stimulation with one or more activators.

Secondary outcomes are the results of the hemostatic tests (e.g., platelet count, factor VIII activity, etc.).

### Statistical analysis

Statistical analysis was performed using IBM SPSS Statistics 25. Normality was tested using the Shapiro-Wilk test and visually assessing the histograms. Normally distributed parameters are displayed as mean ± standard deviation (SD) and nonparametric variables are displayed as the median and interquartile range (IQR). Categorical data are reported as incidences. Missing data were handled using pairwise deletion.

Subgroup analyses were performed in the following groups: children (0–18 years) versus adults and low (0.1–20 mg/kg/day) versus high dosage VPA (>20 mg/kg/day). The two-sample unpaired t-test was used to compare means for parametric variables. Nonparametric variables were compared using the Mann-Whitney U test. Equality of categorical data was tested using either the Pearson Chi-Square test if there were more than 5 cases or the two-sided Fisher’s Exact Test. A correlation was assessed using Pearson’s ρ. P-values <0.05 were considered statistically significant.

## Results

### Patient characteristics

A total of 73 patients was enrolled in this study. The mean age was 33.8 years (range 1–76 years) and 45 patients (61.1%) were male (Table 1). The indication for the use of VPA was epilepsy for almost all patients and the mean dosage was 20.1 mg/kg/day. A majority of the patients received poly-drug therapy since 61 patients (83.6%) used another AED additional to VPA. One patient used an NSAID and one patient carbasalate calcium, both drugs that are known to interfere with hemostasis. 58 patients (79.5%) had a comorbidity such as a syndrome (Rett syndrome, Generalized Epilepsy with Febrile Seizures Plus, Lennox Gastaut syndrome, West syndrome, Dravet syndrome, and RHOBTB2 syndrome) and/or psychomotor retardation and scoliosis.

**Table 1:** Baseline characteristics of the patients who were tested for a VPA-induced coagulopathy.

Characteristic		Number of patients	Value
**Age (years)**		73	33.8 ± 18.2
**Gender**	**Male**	45	61.1%
** **	**Female**	28	38.4%
**Dosage VPA (ml/kg/day)**		73	20.1 ± 7.2
**Duration of treatment (months)**		11	120.4 ± 128.4
**Indication VPA**	**Epilepsy**	71	97.3%
** **	**Bipolar disorder**	2	2.7%
**Use of other AEDs** ^**1**^		61	83.6%
**Comorbidities**		58	79.5%
** **	**Psychomotor retardation**	14	19.2%
** **	**Scoliosis**	11	15.1%
** **	**Syndrome**	10	13.7%
** **	**Mental disability**	9	12.3%
** **	**Cerebrovascular accident**	3	4.1%
** **	**Other**	11	15.1%
**Family history for coagulopathies** ^**2**^	**Positive**	2	2.7%
** **	**Negative**	5	6.8%
** **	**Unknown**	66	90.4%
**Hb (mmol/L)**		73	8.8 ± 0.97
**Leukocyte count (cells x 10** ^**9**^ **/L)**	**Available**	35	6.1 ± 1.8
	**Unavailable**	38	
**Thrombocyte count (cells x 10** ^**9**^ **/L)**		73	226.4 ± 67.0
**Blood type**	**O+**	28	38.4%
** **	**O-**	2	2.7%
** **	**A+**	27	37.0%
** **	**A-**	2	2.7%
** **	**B+**	7	9.6%
** **	**B-**	1	1.4%
** **	**AB+**	3	4.1%
	**Unavailable**	3	4.1%

Data are expressed as mean ± standard deviation, median (interquartile range), or percentage (number).

Abbreviations: AED = anti-epileptic drug; Hb = hemoglobin; VPA = valproic acid.

^1^ Other AEDs used in this population are the following: carbamazepine, levetiracetam, lamotrigine, topiramate, zonisamide, lacosamide, oxcarbazepine, perampanel, brivaracetam, phenytoin, stiripentol, clonazepam, vigabatrin, clobazam and midazolam.

^2^ Coagulopathy is defined as any abnormality found in laboratory hemostatic tests.

Patients underwent one of the following procedures: resective brain surgery, stereoelectroencephalography (SEEG), implantation of a vagus nerve stimulator (VNS) or replacement of the VNS battery, spinal fusion, correction of hip dysplasia, adenotonsillectomy, or general (abdominal) surgery. No postoperative bleeding complications were reported.

Routine laboratory findings are presented in Table 1.

### Coagulopathies

Laboratory results demonstrated 34 patients (46.6%) had a coagulopathy (Table 2). The most frequently found coagulopathy was a platelet function disorder in 24 patients (36.4%). Thrombocytopenia was found in 7 patients (9.6%). None of the patients presented with severe thrombocytopenia (<50 x 10^9^/L). Other coagulopathies found were hypofibrinogenemia (15.2%), low VWF activity (11.1%; 2 patients with blood type O positive and 2 patients with A positive), factor XIII deficiency (6.7%), prolonged aPTT (10.5%), prolonged PFA-ADP (8.5%) and prolonged PFA-EPI (10.2%). Prolonged PT or a factor VIII deficiency was not found in this population. One patient used an NSAID during the laboratory tests and showed a prolonged PFA-ADP and PFA-EPI. Another patient used carbasalate calcium and had a platelet function disorder, which dissolved 7 days after discontinuation of the drug. Six patients with a coagulopathy, VPA was tapered and coagulation tests were performed again after 4 weeks. In 3/6 of the patients (50.0%) laboratory tests normalized. Average values of all laboratory assays are presented as supplementary data in S2 Table. Some tests were only performed in a subgroup, therefore the number of patients is displayed for all parameters.

**Table 2:** Incidence of the different coagulopathies that were found in patients using VPA.

Type of coagulopathy		Incidence
**Coagulopathy** ^**1**^		46.6% (34/73)
**Normalized test results >4 weeks after discontinuation of VPA**		50.0% (3/6)
**Thrombocytopenia**	**Total**	9.6% (7/73)
** **	**Platelet count 50–99 cells x 10** ^**9**^ **/L**	2.7% (2/73)
** **	**Platelet count 100–149 x cells 10** ^**9**^ **/L**	6.8% (5/73)
**Prolonged aPTT**		10.5% (4/38)
**Prolonged PT**		0.0% (0/36)
**Hypofibrinogenemia**		15.2% (5/33)
**Low VWF activity**		11.1% (4/36)
**fVIII deficiency**		0.0% (0/32)
**fXIII deficiency**		6.7% (2/30)
**Prolonged PFA-ADP**		8.5% (5/59)
**Prolonged PFA-EPI**		10.2% (6/59)
**Platelet function disorder** ^**2**^		36.4% (24/66)

The different coagulopathies are defined as laboratory results below or above the hospital reference range. Data are expressed as percentages (number of patients).

Abbreviations: ADP = adenosine diphosphate; aPTT = activated partial prothrombin time; EPI = epinephrine; fVIII = factor VIII; fXIII = factor XIII; PFA = platelet function analyzer; PT = prothrombin time; VPA = valproic acid; VWF = von Willebrand factor.

^1^ Coagulopathy is defined as any abnormality found in laboratory hemostatic tests.

^2^ Platelet function disorder is defined as one or more abnormal test results in an agonist panel of light transmission aggregometry. One patient used a non-steroidal anti-inflammatory drug and one patient carbasalate calcium.

### LTA

LTA was performed in 66 patients. An abnormal LTA result (maximal aggregation below reference values) was found in 36.4% of the patients using VPA (Table 2). Test results of COL 1 μg/mL were abnormal in 18 patients (27.3%) and EPI μmol/mL in 12 patients (18.2%) (Table 3). However, with COL 4 μg/mL and RIST 1.5 mg/mL aggregations were never decreased. Due to missing data, patient numbers of the descriptive and numeral results differ.

**Table 3:** Results of LTA (maximal aggregation) to test the function of the thrombocytes by activating the different receptors with AA, TRAP, COL, ADP, and EPI in patients who use valproic acid.

LTA	n	Value	Incidence
**LTA-AA (%)**	66	86.0 (79.0–92.0)	1.5% (1) negative
			1.5% (1) normal/delayed
**LTA-TRAP (%)**	65	83.0 (78.0–89.0)	1.5% (1) decreased/reversible
**LTA-COL 1 (%)**	63	82.8 ± 7.7	10.6% (7) negative
			12.1% (8) decreased
			4.5% (3) slightly decreased
**LTA-COL 4 (%)**	64	75.0 (55.0–81.0)	0.0% (0) abnormal
**LTA-RIST (%)**	63	87.0 (79.0–89.0)	0.0% (0) abnormal
**LTA-ADP 5 (%)**	64	77.5 (71.0–85.0)	1.5% (1) decreased
			4.5% (3) slightly decreased
			1.5% (1) reversible
**LTA-ADP 10 (%)**	61	80.1 ± 8.8	1.6% (1) decreased and reversible
**LTA-EPI (%)**	64	81.0 (73.0–86.0)	7.6% (5) negative
			6.1% (4) decreased
			3.0% (2) normal/delayed
			1.5% (1) delayed/delayed second wave

Data are expressed as mean ± standard deviation, median (interquartile range) or percentage (number). LTA test results: normal = 60–100%, slightly decreased = 45–60%, decreased = 15–45%, negative = <15%.

Abbreviations: AA = arachidonic acid 1 mmol/L; ADP-5 = adenosine diphosphate 5 μmol/L; ADP-10 = adenosine diphosphate 10 μmol/L; COL-1 = collagen 1 μg/mL; COL-4 = collagen 4 μg/mL; EPI = epinephrine; LTA = light transmission aggregometry; n = number of patients tested; RIST = ristocetine; TRAP = thrombin receptor activating peptide.

**Fig 1:**
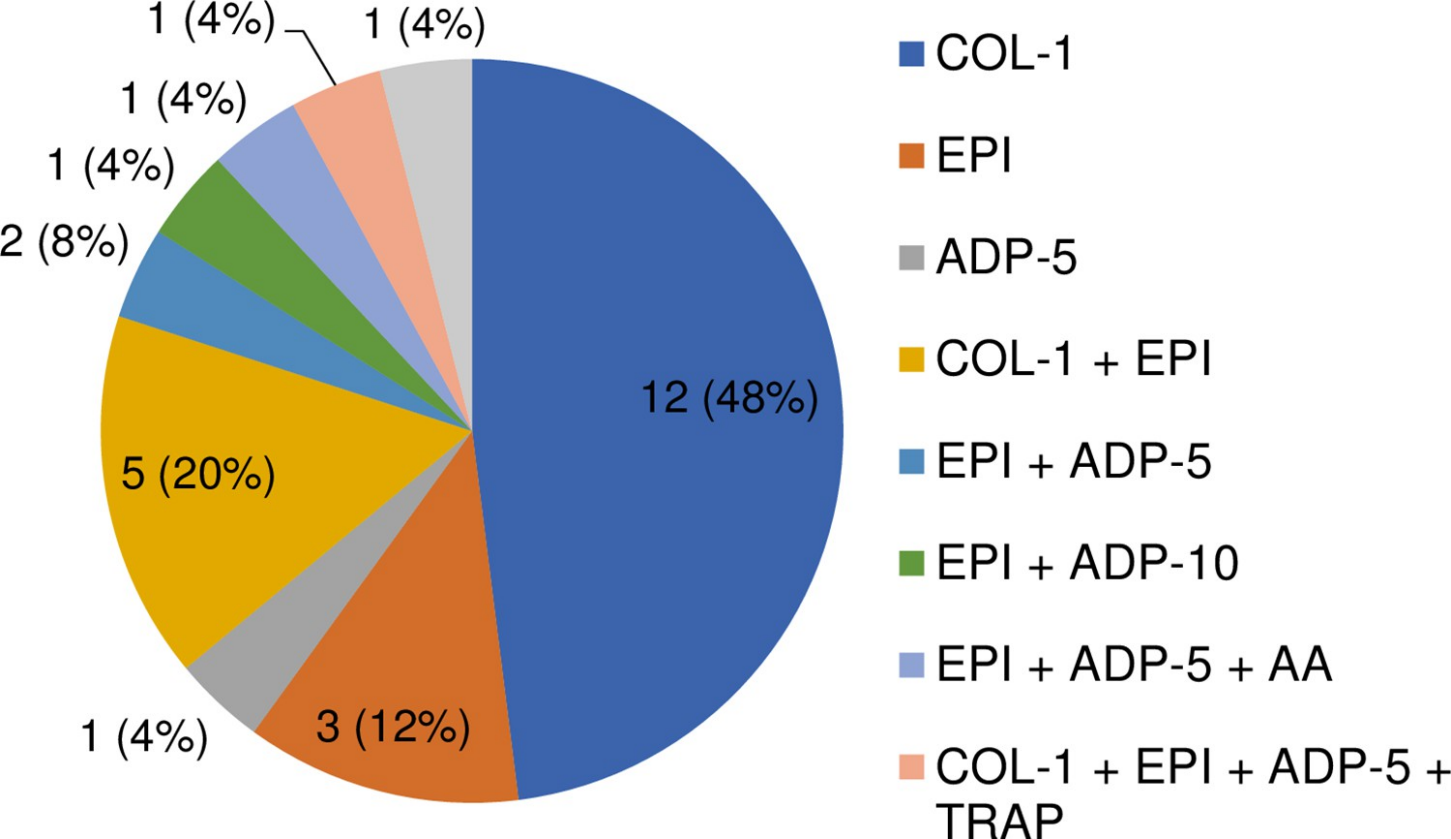
Number of patients with the different combinations of abnormalities in LTA (maximal aggregation) using valproic acid. Abbreviations: AA = arachidonic acid 1 mmol/L; ADP-5 = adenosine diphosphate 5 μmol/L; ADP-10 = adenosine diphosphate 10 μmol/L; COL-1 = collagen 1 μg/mL; COL-4 = collagen 4 μg/mL; EPI = epinephrine; LTA = light transmission aggregometry; RIST = ristocetine; TRAP = thrombin receptor activating peptide.

Fig 1 shows the incidence of the combination of LTA-associated abnormalities. Besides exclusive defects in COL 1 μg/mL and EPI 10 μmol/L, 5 patients showed a combined decreased reaction on COL 1 μg/mL and EPI 10 μmol/L. 1 patient had an abnormal test result with TRAP 15 μmol/mL, although abnormalities were also found by testing with COL 1 μg/mL, EPI 10 μmol/L and ADP 5 μmol/L in this patient. There was also 1 patient with a decreased reaction on ADP-10 μmol/L, which was combined with an abnormal test result with EPI 10 μmol/L.

### Subgroup analysis children versus adults

Twenty children and 53 adults were analyzed. In the adult group, the percentage of males was significantly higher, but there was no significant difference in mean dosage and the use of other AEDs (Table 4). Data suggest that children were more frequently diagnosed with a coagulopathy than adults, respectively 65.0% and 39.6% (p-value 0.053).

**Table 4:** Subgroup analysis of children (0–18 years) versus adults (>18 years) using VPA. Baseline characteristics and the different laboratory hemostatic test results are shown.

		Children		Adults		*p-value*
		n	Value	n	Value	
**Age (years)**		20	12.0 ± 5.0	53	42.0 ± 14.0	<0.001*
**Gender**	**Male**	8/20	40.0%	37/53	70.0%	0.019^†^
** **	**Female**	12/20	60.0%	16/53	30.0%	
**Dosage (ml/kg/day)**		20	22.3 ± 6.6	53	19.2 ± 7.3	0.098*
**Other AEDs**		14/20	70.0%	47/53	88.7%	0.055^†^
**Coagulopathy** ^**1**^		13/20	65.0%	21/53	39.6%	0.053^†^
**Normalized test results >4 weeks after discontinuation of VPA**		0	0.0%	3/6	50.0%	n.a.
**Thrombocytopenia**		1/20	5.0%	6/53	11.3%	0.665^¶^
**Hypofibrinogenemia**		5/14	35.7%	0/19	0.0%	0.008^¶^
**Low VWF activity**		3/15	20.0%	1/21	4.8%	0.287^¶^
**fVIII deficiency**		0/15	0.0%	0/17	0.0%	n.a.
**fXIII deficiency**		2/12	16.7%	0/18	0.0%	0.152^¶^
**Prolonged aPTT**		1/15	6.7%	3/23	13.0%	1.000^¶^
**Prolonged PT**		0/15	0.0%	0/21	0.0%	n.a.
**Prolonged PFA-ADP**		3/12	25.0%	2/47	4.3%	0.052^¶^
**Prolonged PFA-EPI**		2/12	16.7%	4/47	8.5%	0.591^¶^
**Platelet function disorder** ^**2**^		8/14	57.1%	16/52	24.2%	0.069^†^

The different coagulopathies are defined as laboratory results below or above the hospital reference range.

Data are expressed as mean ± standard deviation, median (interquartile range), or percentage (number).

Abbreviations: AED = anti-epileptic drug; ADP = adenosine diphosphate; aPTT = activated partial prothrombin time; EPI = epinephrine; fVIII = factor VIII; fXIII = factor XIII; Hb = hemoglobin; n = number of patients; n.a. = not applicable; PFA = platelet function analyzer; PT = prothrombin time; VPA = valproic acid; VWF = von Willebrand factor.

* Two-sample unpaired t-test

† Pearson Chi-Square

‡ Mann-Whitney U test

¶ Fisher’s Exact Test (2-sided)

^1^ Coagulopathy is defined as any abnormality found in laboratory hemostatic tests.

^2^ Platelet function disorder is defined as one or more abnormal test results in an agonist panel of light transmission aggregometry. One patient used a non-steroidal anti-inflammatory drug and one patient carbasalate calcium.

**Fig 2:**
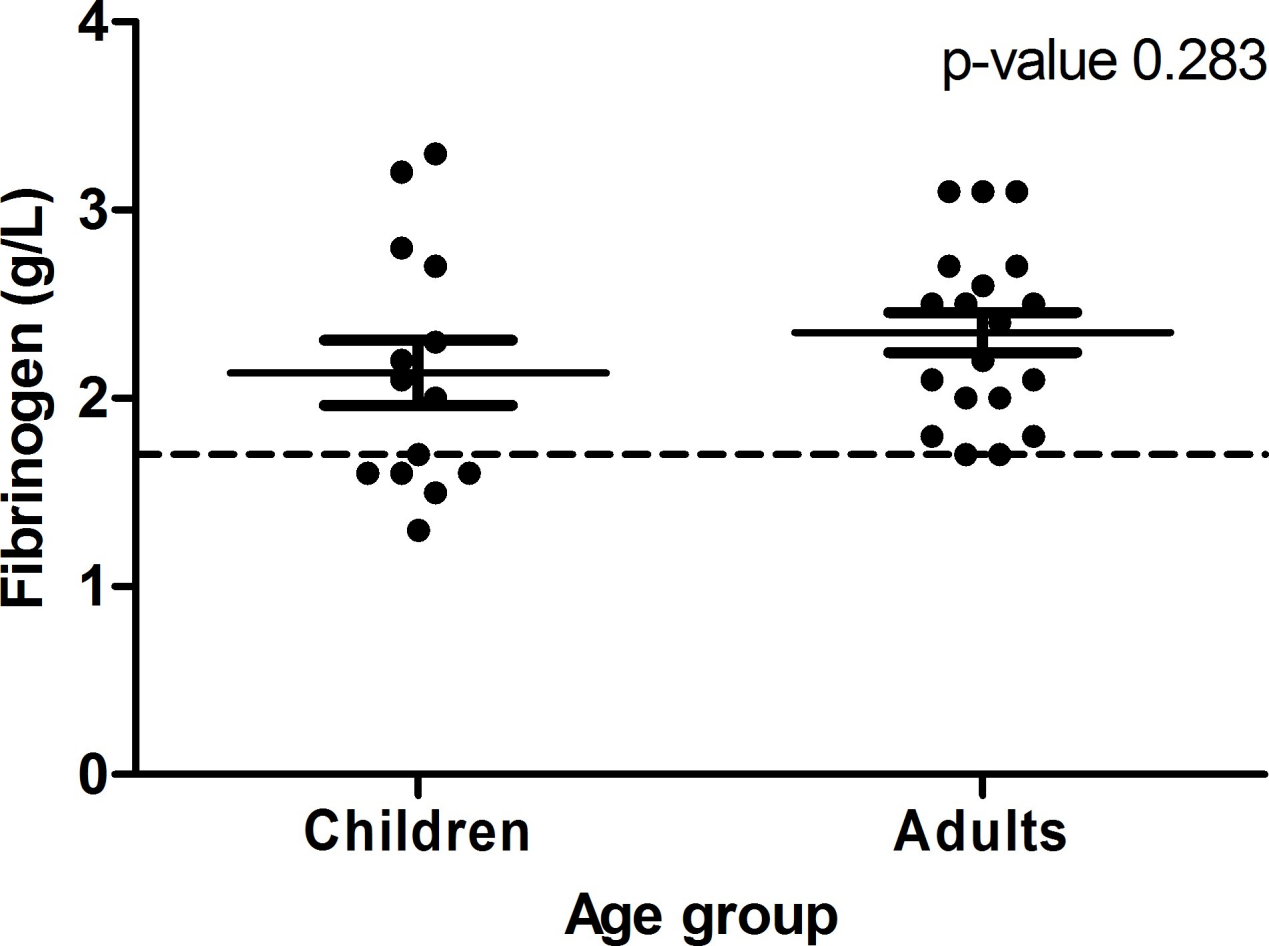
Level of fibrinogen (g/L) in children and adults using valproic acid. The bars represent the mean value and standard variation. Mean values did not differ significantly (p-value 0.283).

Hypofibrinogenemia (n = 5) was exclusively found in children (Fig 2; not statistically significant). Although not statistically significant, platelet function disorder occurred twice as often in children compared to adults (respectively 57.1% and 24.2%, p-value 0.069). Supplementary data of the laboratory results can be found in S3 Table.

### Subgroup analysis low versus high dosage VPA

Patients with a low (0.1–20 mg/kg/day; n = 41) and a high VPA dosage (>20 mg/kg/day; n = 32) were compared, since the therapeutic dosage is 20–30 mg/kg/day according to local policy. Baseline characteristics (mean age, percentage male, and prescription of other AED) did not differ significantly between groups (Table 5).

**Table 5:** Subgroup analysis of patients with a low dosage (0.1–20 mg/kg/day) versus a high dosage VPA (>20 mg/kg/day). Baseline characteristics and the different hemostatic test results are shown.

		Low dosage VPA		High dosage VPA		*p-value*
		n	Value	n	Value	
**Age (years)**		41	37.1 ± 17.8	32	29.6 ± 18.1	0.081*
**Gender**	**Male**	27/41	65.9%	18/32	56.3%	0.402^†^
** **	**Female**	14/41	34.1%	14/32	43.8%	
**Dosage (ml/kg/day)**		41	14.6 ± 3.5	32	27.0 ± 4.0	<0.001*
**Other AEDs**		34/41	82.9%	27/32	84.4%	0.868^†^
**Coagulopathy** ^**1**^		18/41	43.9%	16/32	50.0%	0.604^†^
**Normalized test results >4 weeks after discontinuation of VPA**		2/4	50.0%	1/2	50.0%	1.000^¶^
**Thrombocytopenia**		3/41	7.3%	4/32	12.5%	0.692^¶^
**Hypofibrinogenemia**		1/17	5.9%	4/16	25.0%	0.175^¶^
**Low VWF activity**		2/19	10.5%	2/17	11.8%	1.000^¶^
**fVIII deficiency**		0/17	0.0%	0/15	0.0%	n.a.
**fXIII deficiency**		1/15	6.7%	1/15	6.7%	1.000^¶^
**Prolonged aPTT**		2/19	10.5%	2/19	10.5%	1.000^¶^
**Prolonged PT**		0/18	0.0%	0/18	0.0%	n.a.
**Prolonged PFA-ADP**		3/36	8.3%	2/23	8.7%	1.000^¶^
**Prolonged PFA-EPI**		5/36	13.9%	1/23	4.3%	0.389^¶^
**Platelet function disorder** ^**2**^		14/37	37.8%	10/29	34.5%	0.779^†^

The different coagulopathies are defined as laboratory results below or above the hospital reference range.

Data are expressed as mean ± standard deviation, median (interquartile range) percentage (number).

Abbreviations: AED = anti-epileptic drug; ADP = adenosine diphosphate; aPTT = activated partial prothrombin time; EPI = epinephrine; fVIII = factor VIII; fXIII = factor XIII; Hb = hemoglobin; n = number of patients; n.a. = not applicable; PFA = platelet function analyzer; PT = prothrombin time; VPA = valproic acid; VWF = von Willebrand factor.

* Two-sample unpaired t-test

† Pearson Chi-Square

‡ Mann-Whitney U test

¶ Fisher’s Exact Test (2-sided)

^1^ Coagulopathy is defined as any abnormality found in laboratory hemostatic tests.

^2^ Platelet function disorder is defined as one or more abnormal test results in an agonist panel of light transmission aggregometry. 1 patient used a non-steroidal anti-inflammatory drug and 1 patient carbasalate calcium.

**Fig 3:**
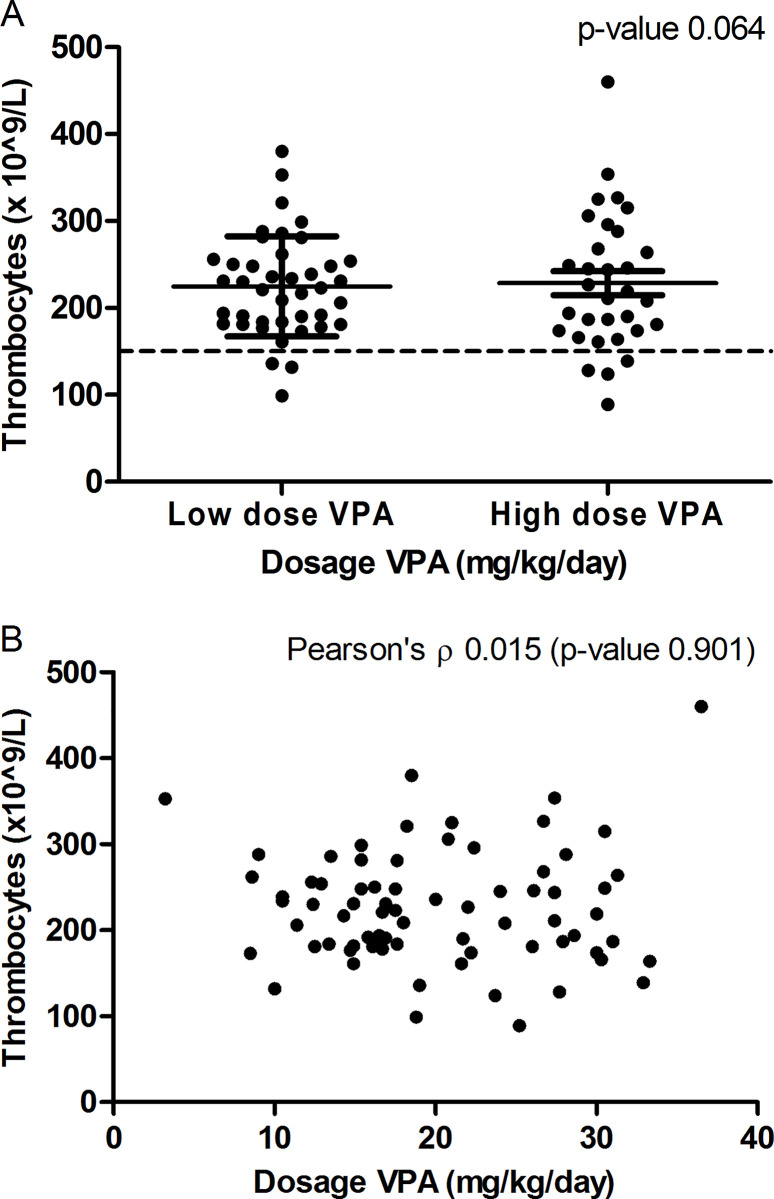
a. Graph of the platelet count (x 10^9^/L) in patients in the low dosage (0.1–20 mg/kg/day) and high dosage (>20 mg/kg/day) valproic acid (VPA) group. Bars are representing the mean value and standard deviation. Mean values did not differ significantly (p-value 0.064). b. Scatter plot of the dosage VPA (mg/kg/day) versus the platelet count (x 10 /L) (Pearson’s ρ 0.015, p-value 0.901).

This subgroup analysis did not show any significant difference between the low and high dosage group in the incidence of VPA-induced coagulopathies (complete data of the laboratory results are presented as supplementary data in S4 Table). Three patients (7.3%) in the low dosage group compared to 4 patients (12.5%) in the high dosage group were diagnosed with thrombocytopenia (p-value 0.501) (Fig 3A). No correlation was present between platelet count and drug dosage, as shown by scatter plot analysis and Pearson’s ρ test (Fig 3B).

## Discussion

VPA is a commonly prescribed anti-epileptic drug in children and adults. Because of the severity of epilepsy, it is often undesirable or impossible to taper off the VPA. Since the launch of VPA, there are concerns about adverse effects on hemostasis, although the literature is conflicting and the pathophysiology is still poorly understood [13, 14, 17–21]. This study showed that almost half of the patients using VPA has, according to our definition, a coagulopathy (46.6%), in particular a platelet function disorder. However, causality was not proven.

To our knowledge, only 2 studies have reported LTA results in respectively 29 and 20 patients using VPA. In contrast to our results, Serdaroglu et al. reported normal platelet function. Unfortunately, a comparison with our results is hampered because the applied concentration of the platelet agonists is not described [8, 22]. Likewise, Zighetti et al. showed that values did not differ between patients using VPA and controls, performing LTA with COL 2 μg/mL, ADP 2 and 4 μmol/mL, U46619 1 μg/mL and TRAP-6 10 μg/mL [22]. More reports have been published about testing aggregation by using a less sensitive method with a lumi-aggregometer in whole blood, though results between studies remain conflicting [23]. Some articles reported no difference in aggregation compared to patients without VPA [24, 25]. A few others reported decreased aggregation with COL and ADP in comparison to a control group, which is in concordance with our results. However, none of these studies reported values below the reference range [15, 16, 26]. A hypothesis of the VPA-induced decreased aggregation with COL and ADP might be its relation with reduced exocytosis of ATP release from dense bodies [16].

In our study, patients were diagnosed with a platelet function disorder if one or more LTA assays were abnormal according to local reference values. Results demonstrated that COL 1 μg/mL was most often decreased. The use of the lower COL 1 μg/mL concentration is questioned by the study of Munnix et al, which showed that LTA results varied significantly in healthy volunteers using lower collagen concentrations (0.2–2 μg/mL) as compared to higher concentrations [27]. Indeed, the Scientific and Standardization Committee of the International Society on Thrombosis and Haemostasis (SSC-ISTH) advises performing the LTA assay with COL 2 μg/mL [28]. After standardization almost all laboratories use COL 2 μg/mL, resulting in less abnormal LTA findings [27]. Therefore, it could be argued that in our study the incidence of platelet function disorder could have been lower if COL 2 μg/mL was used instead of 1 μg/mL. Nonetheless, most patients (n = 15, 83.3%) with abnormal COL 1 μg/mL had a severely decreased or negative COL 1 μg/mL aggregation curve, suggesting that even higher collagen concentration would not likely result in fewer abnormal results.

One of the most reported side effects of VPA is thrombocytopenia, which had an incidence of 9.6% in our study [8–12]. None of the patients in our study needed a platelet transfusion for their thrombocytopenia. Two publications described a negative correlation of platelet count with the dosage of VPA (mg/day), although no correlation has been found in our study [11, 26]. However, serum VPA levels were not measured in both the presented articles and our study. Therefore, with these data correlation between serum level VPA and platelet count remains unknown as serum levels VPA are unpredictable in patients with the same dosage per kilogram [29]. Though in three articles using serum VPA levels, the same correlation as previously described has been found [12, 26, 30].

Another coagulopathy found in our patient population was hypofibrinogenemia. This was only observed in children (p-value 0.008) and this result would not have been affected if we applied reference ranges from Appel et al. [31] The result is comparable to previous studies which demonstrated a hypofibrinogenemia in 8.3 to 57% of the children with VPA, whereas it was only found in 4.0% of the adult patients [8, 9, 18, 25, 32, 33].

Moreover, data suggested that the incidence of coagulopathies in general was almost twice as high in children compared to adults. Since the dosage VPA did not differ significantly between the adult and pediatric groups, it is unlikely that this explains the difference. A possible explanation could be that the hemostatic profile changes with aging [34]. However, Appel et al. showed that differences were more pronounced in results of hemostatic assays in children below 1 year of age, but PT and aPTT differed statistically significantly between age groups. Nonetheless, no patients had a prolonged PT, and only one child aged 14 years had a prolonged aPTT (108 sec) that was above the age-dependent reference range of Appel et al. [31]. Because the age in our study population ranged from 1 to 76 years, we chose to adhere to hospital reference ranges which were not corrected for age, except for Hb, H,t and leukocyte count. The higher incidence of coagulopathies in children found in our study might be influenced by applying the uniform reference values.

A limitation of our study was that not all laboratory tests were performed in the whole cohort due to the retrospective character of this study, not enough blood that could be collected, and protocol extension. Furthermore, besides no postoperative bleeding events, further clinical outcome is not assessed in our study. Even though, this was not the goal of our study. The frequency of bleeding events, morbidity, and mortality remains unclear in the literature as well [17–21]. Another limitation of this study is the provability of VPA-induced coagulopathy since most patients were deemed unable to discontinue VPA to repeat laboratory hemostatic testing. A major percentage of the patients also used other AEDs, which could have affected our results. However, a review of Verrotti et al. showed that other AEDs used in our study population specifically decrease platelet count and do not induce other hemostatic abnormalities [35]. From this we conclude that the concomitant use of other AEDs in our patient group should not have influenced the results besides the platelet count, although further studies are needed to confirm this. Another possible predictor of coagulopathies is the presence of a syndrome, as literature illustrated that some syndromes, e.g. Noonan syndrome, are associated with bleeding disorders [36]. Nonetheless, we did not find publications about the association of coagulopathies with the specific syndromes in our study population.

This is to our current knowledge the first study on extensive hematological laboratory testing in a relatively large cohort of 73 pediatric and adult patients with VPA scheduled for elective high-risk surgeries. In addition, platelet aggregation was tested with the gold standard technique (LTA), whereas most other publications reported thrombocyte function assessed using lumi-aggregometer which is less sensitive in detecting mild platelet disorders [23].

Our data demonstrated that a considerable number of patients using VPA in a therapeutic dosage has a coagulopathy according to our local reference ranges, although causality was not proven. More research, preferentially in a prospective format, is needed to prove that coagulopathies are induced by VPA, to study cost-effectiveness, to develop a “standard” set of preoperative laboratory tests in case of VPA usage, and to assess clinical outcome (e.g., blood loss perioperative, morbidity and mortality).

## Supporting information

S1 Table Reference ranges of laboratory tests. Abbreviations: aPTT = activated partial prothrombin time; fVIII = factor VIII; fXIII = factor XIII; Hb = hemoglobin; Ht = hematocrit; LTA = light transmission aggregometry; PFA-EPI = platelet function analyzer with collagen and epinephrine; PFA-ADP = platelet function analyzer with collagen and adenosine diphosphate; PT = prothrombin time; VWF = von Willebrand factor.
(DOCX)

S2 Table Laboratory test results of patients using valproic acid. Data are expressed as mean ± standard deviation, median (interquartile range) or percentage (number). Abbreviations: AA = arachidonic acid 1 mmol/L; ADP-5 = adenosine diphosphate 5 μmol/mL; ADP-10 = adenosine diphosphate 10 μmol/mL; aPTT = activated partial prothrombin time; COL-1 = collagen 1 μg/mL; COL-4 = collagen 4 μg/mL; EPI = epinephrine; fVIII = factor VIII; fXIII = factor XIII; Hb = hemoglobin; Ht = hematocrit; LTA = light transmission aggregometry; n = number of patients tested; PFA = platelet function analyzer; PT = prothrombin time; RIST = ristocetine; TRAP = thrombin receptor activating peptide; VWF = von Willebrand factor.
(DOCX)

S3 Table Laboratory test results of the subgroup analysis of children (0–18 years) versus adults (>18 years) using valproic acid. Data are expressed as mean ± standard deviation, median (interquartile range) or percentage (number). Abbreviations: AA = arachidonic acid 1 mmol/L; ADP-5 = adenosine diphosphate 5 μmol/mL; ADP-10 = adenosine diphosphate 10 μmol/mL; aPTT = activated partial prothrombin time; COL-1 = collagen 1 μg/mL; COL-4 = collagen 4 μg/mL; EPI = epinephrine; fVIII = factor VIII; fXIII = factor XIII; LTA = light transmission aggregometry; n = number of patients tested; PFA = platelet function analyzer; PT = prothrombin time; RIST = ristocetine; TRAP = thrombin receptor activating peptide; VPA = valproic acid; VWF = von Willebrand factor. * Two sample unpaired t-test, ‡ Mann-Whitney U test.
(DOCX)

S4 Table Laboratory test results of the subgroup analysis of coagulopathies in low dosage (0.1–20 mg/kg/day) versus high dosage valproic acid (>20 mg/kg/day). Data are expressed as mean ± standard deviation, median (interquartile range) or percentage (number). Abbreviations: AA = arachidonic acid 1 mmol/L; ADP-5 = adenosine diphosphate 5 μmol/mL; ADP-10 = adenosine diphosphate 10 μmol/mL; aPTT = activated partial prothrombin time; COL-1 = collagen 1 μg/mL; COL-4 = collagen 4 μg/mL; EPI = epinephrine; fVIII = factor VIII; fXIII = factor XIII; LTA = light transmission aggregometry; n = number of patients tested; PFA = platelet function analyzer; PT = prothrombin time; RIST = ristocetine; TRAP = thrombin receptor activating peptide; VPA = valproic acid; VWF = von Willebrand factor. * Two sample unpaired t-test, ‡ Mann-Whitney U test.
(DOCX)
