# Supplementary material for: Assessment of need for hemostatic evaluation in patients taking valproic acid: A retrospective cross-sectional study
Source: PLoS One. 2022 Feb 25;17(2):e0264351. doi: 10.1371/journal.pone.0264351 (PMC8880909; doi:10.1371/journal.pone.0264351)
Supplement: S1 Table — Abbreviations: aPTT = activated partial prothrombin time; fVIII = factor VIII; fXIII = factor XIII; Hb = hemoglobin; Ht = hematocrit; LTA = light transmission aggregometry; PFA-EPI = platelet function analyzer with collagen and epinephrine; PFA-ADP = platelet function analyzer with collagen and adenosine diphosphate; PT = prothrombin time; VWF = von Willebrand factor. (DOCX) [file pone.0264351.s001.docx]

**S1 Table. Reference ranges of laboratory tests.**

Abbreviations: aPTT = activated partial prothrombin time; fVIII = factor VIII; fXIII = factor XIII; Hb = hemoglobin; Ht = hematocrit; LTA = light transmission aggregometry; PFA-EPI = platelet function analyzer with collagen and epinephrine; PFA-ADP = platelet function analyzer with collagen and adenosine diphosphate; PT = prothrombin time; VWF = von Willebrand factor.

| **Laboratory test** | | **Reference range** |
| --- | --- | --- |
| **Hb** | **0.5-2 years** | 6.8-8.1 mmol/L |
|  | **2-8 years** | 7.5-8.7 mmol/L |
|  | **8-15 years** | 7.1-9.0 mmol/L |
|  | **Male >15 years** | 8.2-11.0 mmol/L |
|  | **Female >15 years** | 7.3-9.7 mmol/L |
| **Ht** | **0.5-2 years** | 0.34-0.46 L/L |
|  | **2-8 years** | 0.36-0.44 L/L |
|  | **8-15 years** | 0.37-0.45 L/L |
|  | **Male >15 years** | 0.41-0.52 L/L |
|  | **Female >15 years** | 0.36-0.48 L/L |
| **Leukocyte count** | **0.5-2 years** | 6.0-18.0 x 10^9^/L |
|  | **2-8 years** | 5.0-15.0 x 10^9^/L |
|  | **8-15 years** | 4.3-13.5 x 10^9^/L |
|  | **>15 years** | 3.5-11.0 x 10^9^/L |
| **Thrombocyte count** | | 150-350 x 10^9^/L |
| **MPV** | | 9.2-12.7 fL |
| **aPTT** | | 23-32 sec |
| **PT** | | 9.9-12.4 sec |
| **Fibrinogen** | | 1.7-4.0 g/L |
| **VWF activity and antigen** | | 50-150% |
| **Ratio VWF activity/antigen** | | >0.7 |
| **fVIII activity** | | 50-200% |
| **fXIII activity** | | 70-140% |
| **PFA-EPI** | | 84-160 sec |
| **PFA-ADP** | | 71-118 sec |
| **LTA** | **Normal** | 60-100% |
|  | **Mild reduced** | 45-60% |
|  | **Reduced** | 15-45% |
|  | **Negative** | <15% |
