# Supplementary material for: Assessment of need for hemostatic evaluation in patients taking valproic acid: A retrospective cross-sectional study
Source: PLoS One. 2022 Feb 25;17(2):e0264351. doi: 10.1371/journal.pone.0264351 (PMC8880909; doi:10.1371/journal.pone.0264351)
Supplement: S2 Table — Data are expressed as mean ± standard deviation, median (interquartile range) or percentage (number). Abbreviations: AA = arachidonic acid 1 mmol/L; ADP-5 = adenosine diphosphate 5 μmol/mL; ADP-10 = adenosine diphosphate 10 μmol/mL; aPTT = activated partial prothrombin time; COL-1 = collagen 1 μg/mL; COL-4 = collagen 4 μg/mL; EPI = epinephrine; fVIII = factor VIII; fXIII = factor XIII; Hb = hemoglobin; Ht = hematocrit; LTA = light transmission aggregometry; n = number of patients tested; PFA = platelet function analyzer; PT = prothrombin time; RIST = ristocetine; TRAP = thrombin receptor activating peptide; VWF = von Willebrand factor. (DOCX) [file pone.0264351.s002.docx]

**S2 Table. Laboratory test results of patients using valproic acid.**

| **Laboratory test** | **n** | **Value** |
| --- | --- | --- |
| **Thrombocyte count (x10^9^/L)** | 73 | 226.4 ± 67.0 |
| **MPV (fL)** | 31 | 10.2 ± 0.8 |
| **aPTT (sec)** | 38 | 29.0 (27.0-30.0) |
| **PT (sec)** | 36 | 11.2 ± 0.5 |
| **Fibrinogen (g/L)** | 33 | 2.3 ± 0.6 |
| **VWF activity (%)** | 36 | 94.9 ± 37.6 |
| **VWF antigen (%)** | 35 | 92.3 ± 33.4 |
| **fVIII activity (%)** | 32 | 114.4 ± 42.0 |
| **fXIII activity (%)** | 30 | 101.5 (80.0-107.0) |
| **PFA-ADP (sec)** | 59 | 86.0 (74.0-96.0) |
| **PFA-EPI (sec)** | 59 | 114.0 (99.0-132.0) |
| **LTA-AA (%)** | 64 | 86.0 (79.0-92.0) |
| **LTA-TRAP (%)** | 65 | 83.0 (78.0-89.0) |
| **LTA-COL 1 (%)** | 63 | 82.8 ± 7.7 |
| **LTA-COL 4 (%)** | 64 | 75.0 (55.0-81.0) |
| **LTA-RIST (%)** | 63 | 87.0 (79.0-89.0) |
| **LTA-ADP 5 (%)** | 64 | 77.5 (71.0-85.0) |
| **LTA-ADP 10 (%)** | 61 | 80.1 ± 8.8 |
| **LTA-EPI (%)** | 64 | 81.0 (73.0-86.0) |

Data are expressed as mean ± standard deviation, median (interquartile range) or incidence (percentage). Abbreviations: AA = arachidonic acid; ADP-5 = adenosine diphosphate 5 μmol/L; ADP-10 = adenosine diphosphate 10 μmol/L; aPTT = activated partial prothrombin time; COL-1 = collagen 1 μg/mL; COL-4 = collagen 4 μg/mL; EPI = epinephrine; fVIII = factor VIII; fXIII = factor XIII; Hb = hemoglobin; Ht = hematocrit; LTA = light transmission aggregometry; n = number of patients tested; PFA = platelet function analyzer; PT = prothrombin time; RIST = ristocetine; TRAP = thrombin receptor activating peptide; VWF = von Willebrand factor.
