# Supplementary material for: Assessment of need for hemostatic evaluation in patients taking valproic acid: A retrospective cross-sectional study
Source: PLoS One. 2022 Feb 25;17(2):e0264351. doi: 10.1371/journal.pone.0264351 (PMC8880909; doi:10.1371/journal.pone.0264351)
Supplement: S3 Table — Data are expressed as mean ± standard deviation, median (interquartile range) or percentage (number). Abbreviations: AA = arachidonic acid 1 mmol/L; ADP-5 = adenosine diphosphate 5 μmol/mL; ADP-10 = adenosine diphosphate 10 μmol/mL; aPTT = activated partial prothrombin time; COL-1 = collagen 1 μg/mL; COL-4 = collagen 4 μg/mL; EPI = epinephrine; fVIII = factor VIII; fXIII = factor XIII; LTA = light transmission aggregometry; n = number of patients tested; PFA = platelet function analyzer; PT = prothrombin time; RIST = ristocetine; TRAP = thrombin receptor activating peptide; VPA = valproic acid; VWF = von Willebrand factor. * Two sample unpaired t-test, ‡ Mann-Whitney U test. (DOCX) [file pone.0264351.s003.docx]

**S3 Table. Laboratory test results of the subgroup analysis of children (0-18 years) versus adults (>18 years) using valproic acid.**

Data are expressed as mean ± standard deviation, median (interquartile range) or incidence (percentage). Abbreviations: AA = arachidonic acid; ADP-5 = adenosine diphosphate 5 μmol/L; ADP-10 = adenosine diphosphate 10 μmol/L; aPTT = activated partial prothrombin time; COL-1 = collagen 1 μg/mL; COL-4 = collagen 4 μg/mL; EPI = epinephrine; fVIII = factor VIII; fXIII = factor XIII; LTA = light transmission aggregometry; n = number of patients tested; PFA = platelet function analyzer; PT = prothrombin time; RIST = ristocetine; TRAP = thrombin receptor activating peptide; VPA = valproic acid; VWF = von Willebrand factor.

* Two sample unpaired t-test

‡ Mann-Whitney U test

| **Laboratory test** | | **Children** | | **Adults** | | ***p-value*** |
| --- | --- | --- | --- | --- | --- | --- |
|  |  | **n** | **Value** | **n** | **Value** |  |
| **Thrombocyte count (cells x 10^9^/L)** | | 20 | 252 ± 84.4 | 53 | 216.8 ± 57.1 | 0.045^*^ |
| **MPV (fL)** | | 13 | 10.3 ± 0.9 | 18 | 10.1 ± 0.8 | 0.367_*_ |
| **Leukocyte count (cells x 10^9^/L)** | | 16 | 5.7 ± 1.6 | 19 | 6.4 ± 2.0 | 0.285^*^ |
| **aPTT (sec)** | | 15 | 29.0 (28.0-31.0) | 23 | 28.0 (27.0-30.0) | 0.342^‡^ |
| **PT (sec)** | | 15 | 11.4 ± 0.5 | 21 | 11.0 ± 0.4 | 0.014^*^ |
| **Fibrinogen (g/L)** | | 14 | 2.1 ± 0.6 | 19 | 11.0 ± 0.4 | 0.014^*^ |
| **VWF activity (%)** | | 15 | 82.7 ± 29.9 | 21 | 103.6 ± 40.7 | 0.100^*^ |
| **VWF antigen (%)** | | 15 | 80.8 ± 27.8 | 20 | 100.9 ± 35.3 | 0.079^*^ |
| **fVIII activity (%)** | | 15 | 108.3 ± 46.7 | 17 | 119.8 ± 38.0 | 0.446^*^ |
| **fXIII acitivity (%)** | | 12 | 85.0 (79.0-105.5) | 18 | 104.5 (84.0-127.0) | 0.117^‡^ |
| **PFA-ADP (sec)** | | 12 | 92.0 (76.0-115.5) | 47 | 83.0 (74.0-96.0) | 0.175^‡^ |
| **PFA-EPI (sec)** | | 12 | 119.0 (110.5-145.0) | 47 | 113.0 (98.0-130.0) | 0.314^‡^ |
| **LTA-AA (%)** | | 12 | 87.5 (79.0-91.0) | 52 | 85.0 (79.0-93.0) | 0.750^‡^ |
| **LTA-TRAP (%)** | | 13 | 84.0 (82.0-89.0) | 52 | 83.0 (75.0-88.5) | 0.517^‡^ |
| **LTA-COL 1 (%)** | | 12 | 47.5 (22.0-79.5) | 52 | 75.0 (59.0-82.0) | 0.078^‡^ |
| **LTA-COL 4 (%)** | | 12 | 84.8 ± 8.0 | 51 | 82.3 ± 7.6 | 0.318^*^ |
| **LTA-RIST (%)** | | 12 | 87.0 (85.5-88.5) | 51 | 78.0 (70.0-86.0) | 0.419^‡^ |
| **LTA-ADP 5 (%)** | | 13 | 77.0 (74.0-81.0) | 51 | 78.0 (70.0-86.0) | 0.993^‡^ |
| **LTA-ADP 10 (%)** | | 12 | 79.8 ± 7.1 | 49 | 80.2 ± 9.2 | 0.869^*^ |
| **LTA-EPI (%)** | | 13 | 82.0 (74.0-88.0) | 51 | 81.0 (73.0-86.0) | 0.828^‡^ |
