# Supplementary material for: Assessment of need for hemostatic evaluation in patients taking valproic acid: A retrospective cross-sectional study
Source: PLoS One. 2022 Feb 25;17(2):e0264351. doi: 10.1371/journal.pone.0264351 (PMC8880909; doi:10.1371/journal.pone.0264351)
Supplement: S4 Table — Data are expressed as mean ± standard deviation, median (interquartile range) or percentage (number). Abbreviations: AA = arachidonic acid 1 mmol/L; ADP-5 = adenosine diphosphate 5 μmol/mL; ADP-10 = adenosine diphosphate 10 μmol/mL; aPTT = activated partial prothrombin time; COL-1 = collagen 1 μg/mL; COL-4 = collagen 4 μg/mL; EPI = epinephrine; fVIII = factor VIII; fXIII = factor XIII; LTA = light transmission aggregometry; n = number of patients tested; PFA = platelet function analyzer; PT = prothrombin time; RIST = ristocetine; TRAP = thrombin receptor activating peptide; VPA = valproic acid; VWF = von Willebrand factor. * Two sample unpaired t-test, ‡ Mann-Whitney U test. (DOCX) [file pone.0264351.s004.docx]

**S4 Table. Laboratory test results of the subgroup analysis of coagulation disorders in low dosage (0.1-20 mg/kg/day) versus high dosage valproic acid (>20 mg/kg/day).**

Data are expressed as mean ± standard deviation, median (interquartile range) or incidence (percentage). Abbreviations: AA = arachidonic acid; ADP-5 = adenosine diphosphate 5 μmol/L; ADP-10 = adenosine diphosphate 10 μmol/L; aPTT = activated partial prothrombin time; COL-1 = collagen 1 μg/mL; COL-4 = collagen 4 μg/mL; EPI = epinephrine; fVIII = factor VIII; fXIII = factor XIII; LTA = light transmission aggregometry; n = number of patients tested; PFA = platelet function analyzer; PT = prothrombin time; RIST = ristocetine; TRAP = thrombin receptor activating peptide; VPA = valproic acid; VWF = von Willebrand factor.

* Two sample unpaired t-test

‡ Mann-Whitney U test

| **Laboratory test** | | **Low dosage VPA** | | **High dosage VPA** | | ***p-value*** |
| --- | --- | --- | --- | --- | --- | --- |
|  |  | **n** | **Value** | **n** | **Value** |  |
| **Thrombocyte count (cells x 10^9^/L)** | | 41 | 224.8 ± 57.6 | 32 | 228.4 ± 78.3 | 0.824^*^ |
| **MPV (fL)** | | 15 | 10.0 ± 0.7 | 16 | 10.3 ± 0.9% | 0.403_*_ |
| **Leukocyte count (cells x 10^9^/L)** | | 18 | 6.2 ± 1.6 | 17 | 6.0 ± 2.1 | 0.775^*^ |
| **aPTT (sec)** | | 19 | 28.0 (27.0-30.0) | 19 | 30.0 (28.0-31.0) | 0.210^‡^ |
| **PT (sec)** | | 18 | 11.0 ± 0.5 | 18 | 11.3 ± 0.5 | 0.140^*^ |
| **Fibrinogen (g/L)** | | 17 | 2.4 ± 0.5 | 16 | 2.1 ± 0.5 | 0.064^*^ |
| **VWF activity (%)** | | 19 | 104.8 ± 38.6 | 17 | 83.8 ± 34.2 | 0.094^*^ |
| **VWF antigen (%)** | | 18 | 101.6 ± 35.4 | 17 | 82.4 ± 28.9 | 0.089^*^ |
| **fVIII activity (%)** | | 17 | 116.8 ± 37.5 | 15 | 111.7 ± 47.8 | 0.735^*^ |
| **fXIII acitivity (%)** | | 15 | 105.0 (84.0-118.0) | 15 | 101.0 (78.0-106.0) | 0.418^‡^ |
| **PFA-ADP (sec)** | | 36 | 86.5 (71.5-98.0) | 23 | 84.0 (77.0-96.0) | 0.963^‡^ |
| **PFA-EPI (sec)** | | 36 | 111.0 (95.5-127.5) | 23 | 118.0 (105.0-139.0) | 0.355^‡^ |
| **LTA-AA (%)** | | 36 | 89.0 (79.0-93.0) | 28 | 84.0 (78.5-91.5) | 0.680^‡^ |
| **LTA-TRAP (%)** | | 36 | 83.5 (76.5-88.5) | 29 | 83.0 (79.0-89.0) | 0.838^‡^ |
| **LTA-COL 1 (%)** | | 36 | 75.0 (58.5-81.5) | 28 | 71.0 (27.0-80.0) | 0.405^‡^ |
| **LTA-COL 4 (%)** | | 36 | 82.9 ± 7.4 | 27 | 82.6 ± 8.2 | 0.866^*^ |
| **LTA-RIST (%)** | | 36 | 87.0 (79.0-90.0) | 27 | 85.0 (79.0-89.0) | 0.873^‡^ |
| **LTA-ADP 5 (%)** | | 36 | 77.5 (70.0-84.5) | 28 | 7.5 (72.0-87.5) | 0.432^‡^ |
| **LTA-ADP 10 (%)** | | 35 | 79.6 ± 8.8 | 26 | 80.9 ± 8.8 | 0.588^*^ |
| **LTA-EPI (%)** | | 36 | 80.5 (74.0-85.0) | 28 | 81.5 (71.0-88.0) | 0.542^‡^ |
